# Treating opioid use disorder in veterans with co-occurring substance use: a qualitative study with buprenorphine providers in primary care, mental health, and pain settings

**DOI:** 10.1186/s13722-023-00382-1

**Published:** 2023-05-04

**Authors:** Madeline C. Frost, Elena M. Soyer, Carol E. Achtmeyer, Eric J. Hawkins, Joseph E. Glass, Kevin A. Hallgren, Emily C. Williams

**Affiliations:** 1 Department of Health Systems and Population Health, University of Washington School of Public Health, 1959 NE Pacific St, Seattle, WA 98195 USA; 2 Health Services Research & Development (HSR&D) Center of Innovation for Veteran-Centered and Value-Driven Care, Veterans Affairs (VA) Puget Sound Health Care System, 1660 South Columbian Way, S-152, Seattle, WA 98108 USA; 3 Center of Excellence in Substance Addiction Treatment and Education, Veterans Affairs (VA) Puget Sound Health Care System, 1660 South Columbian Way, Seattle, WA 98108 USA; 4 Department of Psychiatry and Behavioral Sciences, University of Washington School of Medicine, 1959 NE Pacific St, Seattle, WA 98195 USA; 5 Kaiser Permanente Washington Health Research Institute, 1730 Minor Ave, Seattle, WA 98101 USA

**Keywords:** Polysubstance, Comorbidity, Opioid use disorder, Buprenorphine, Veterans, Qualitative

## Abstract

**Background:**

Most people with opioid use disorder (OUD) have co-occurring substance use, which is associated with lower receipt of OUD medications (MOUD). Expanding MOUD provision and care linkage outside of substance use disorder (SUD) specialty settings is a key strategy to increase access. Therefore, it is important to understand how MOUD providers in these settings approach care for patients with co-occurring substance use. This qualitative study of Veterans Health Administration (VA) clinicians providing buprenorphine care in primary care, mental health, and pain settings aimed to understand (1) their approach to addressing OUD in patients with co-occurring substance use, (2) perspectives on barriers/facilitators to MOUD receipt for this population, and (3) support needed to increase MOUD receipt for this population.

**Methods:**

We interviewed a purposive sample of 27 clinicians (12 primary care, 7 mental health, 4 pain, 4 pharmacists) in the VA northwest network. The interview guide assessed domains of the Tailored Implementation for Chronic Diseases Checklist. Interviews were transcribed and qualitatively analyzed using inductive content analysis.

**Results:**

Participants reported varied approaches to identifying co-occurring substance use and addressing OUD in this patient population. Although they reported that this topic was not clearly addressed in clinical guidelines or training, participants generally felt that patients with co-occurring substance use should receive MOUD. Some viewed their primary role as providing this care, others as facilitating linkage to OUD care in SUD specialty settings. Participants reported multiple barriers and facilitators to providing buprenorphine care to patients with co-occurring substance use and linking them to SUD specialty care, including provider, patient, organizational, and external factors.

**Conclusions:**

Efforts are needed to support clinicians outside of SUD specialty settings in providing buprenorphine care to patients with co-occurring substance use. These could include clearer guidelines and policies, more specific training, and increased care integration or cross-disciplinary collaboration. Simultaneously, efforts are needed to improve linkage to specialty SUD care for patients who would benefit from and are willing to receive this care, which could include increased service availability and improved referral/hand-off processes. These efforts may increase MOUD receipt and improve OUD care quality for patients with co-occurring substance use.

## Background

Overdose deaths in the United States are at a record high, [1, 2] and most people with opioid use disorder (OUD) do not receive recommended treatment [3]. There are three approved medications for opioid use disorder (MOUD): buprenorphine, methadone and naltrexone [4]. Buprenorphine and methadone are considered first-line treatment and substantially reduce overdose risk [5–9]. While methadone for OUD must be administered through a federally regulated treatment center, buprenorphine can be prescribed outside of substance use disorder (SUD) specialty settings [4].

The majority of people with OUD use alcohol or other non-opioid drugs [10–13]. Studies have found that patients with co-occurring SUDs are less likely to receive MOUD than those with only OUD [12, 14–16]. Efforts to increase MOUD access have tended to overlook the role of polysubstance use, even though it is highly prevalent among people with OUD and appears to hinder MOUD receipt [10, 17]. As patients may face multiple barriers to accessing care in SUD specialty settings, [18, 19] expanding MOUD provision in other clinical settings is a key strategy to increase access [20, 21]. It is therefore important to understand how clinicians providing buprenorphine care outside of SUD specialty settings approach care for patients with co-occurring substance use, who likely make up a large proportion of their patients with OUD.

Clinical guidelines for OUD treatment state that use of other substances should never be grounds for withholding or suspending MOUD, but that a higher level of care (e.g., SUD specialty care) should be considered for patients actively using other substances, particularly patients with co-occurring SUDs and/or actively using alcohol or sedatives (which may increase risk of respiratory depression while on MOUD) [6, 22]. These guidelines also state that if a higher level of care is not available or acceptable to the patient, this should not prevent or delay the provision of MOUD. The Veterans Health Administration (VA) SUD clinical practice guidelines state that MOUD “should not automatically be discontinued due to a patient’s use of another substance,” and promote the provision of patient-centered OUD care across multiple care settings [5].

Existing research examining providers’ perspectives on this topic is limited. Surveys of U.S. buprenorphine providers (including providers in SUD and non-SUD settings) suggest they may be less likely to prescribe buprenorphine to patients with alcohol or benzodiazepine use disorders relative to those with OUD alone, or may increase monitoring frequency for patients using benzodiazepines [23, 24]. A study that reviewed charts for a random sample of national VA patients with OUD found that clinicians were less likely to recommend MOUD for patients with co-occurring stimulant use disorder [25]. A qualitative study assessing implementation of buprenorphine provision in primary care settings found that most clinics increased monitoring or required additional psychosocial treatment for patients using other substances, and had varying “thresholds” at which they dismissed patients or referred them to specialty SUD care due to other substance use [26]. However, this study was not specifically focused on co-occurring substance use and did not assess providers’ experiences and perceptions that may drive clinical practices. More in-depth qualitative information from clinicians providing buprenorphine care outside of SUD specialty settings is needed to better understand varying approaches to addressing OUD in patients with co-occurring substance use, potential barriers and facilitators to treating them and/or linking them to SUD specialty care, and what support providers need to care for this population.

The VA is the largest OUD treatment provider in the country, [27] over half of VA patients with OUD have co-occurring SUDs, and those with co-occurring SUDs have lower MOUD receipt than those without [12]. Increasing MOUD receipt—including increasing buprenorphine provision outside of SUD specialty settings—is a VA priority, [27, 28] and leaders have called for research that leverages the VA’s status as a learning health system (i.e., an integrated system that can broadly implement and test new interventions and care models) to improve care for patients with OUD and polysubstance use [29]. This qualitative study of VA clinicians providing buprenorphine care in primary care, mental health, and pain settings aimed to understand (1) their approach to addressing OUD in patients with co-occurring substance use, (2) their perspectives on barriers and facilitators to MOUD receipt for this patient population, and (3) support needed to increase MOUD receipt in this population. To our knowledge, this is the first in-depth qualitative study to address barriers and facilitators to MOUD for patients with co-occurring substance use among non-SUD specialist buprenorphine providers.

## Methods

### Study sample and recruitment

We interviewed clinicians in the VA northwest regional network (Veterans Integrated Services Network 20) providing OUD care outside of SUD specialty settings [30]. Clinicians were eligible to participate if (1) they currently provided OUD care outside of an SUD clinic, and (2) they had prescribed buprenorphine for OUD or managed buprenorphine care (e.g., clinical pharmacy specialists, at the time of the study, were unable to prescribe but could manage this care [31]) for ≥ 5 patients. To identify potentially eligible participants, we obtained a list of buprenorphine prescribers in the network from VA Pharmacy Benefits Management Services. The list included clinicians who (1) had a waiver to provide buprenorphine for OUD [32] and (2) had prescribed buprenorphine to ≥ 1 patient within the past 90 days on 2/16/2022. The list excluded one facility that had switched to a new electronic health record system, for which prescribing information was not available. We also employed snowball sampling to expand recruitment beyond this list [33]. Potential participants were sent a recruitment email and up to two follow-up emails. We used purposive sampling to obtain perspectives from different training backgrounds (physician, nurse practitioner/physician assistant, pharmacist), clinical settings (primary care, mental health, pain), VA facility, and clinic rurality. This study was approved by institutional review boards at the University of Washington and VA Puget Sound Healthcare System.

### Data collection

Telephone interviews were conducted from 3/8/2022 to 5/26/2022 by two interviewers with experience in qualitative data collection and researching and/or providing MOUD care (MCF, CEA). The semi-structured interview guide collected clinicians’ training and professional experience through closed-ended questions, and addressed the following topics through open-ended questions: current practices and perspectives related to providing buprenorphine to patients with co-occurring substance use; perception of how training and clinical guidelines address this topic; what factors impact MOUD receipt for VA patients with co-occurring substance use; and what support is needed to increase MOUD receipt for this patient population. The interview guide asked broadly about co-occurring substance use, which may include any use or diagnosed SUDs, in order to allow participants to respond about the type of substance(s) and severity of co-occurring use that was most salient for them. Participants were asked to specify which substance(s) would impact their decision to not provide buprenorphine and/or recommend additional support. The guide was developed to assess domains in the Tailored Implementation for Chronic Diseases (TICD) Checklist, [34, 35] an implementation science tool that organizes factors influencing provision of evidence-based care into categories including individual health professional factors, providers’ perceptions of patient factors, professional interactions, incentives and resources, capacity for organizational change, and social, political and legal factors. Interviews lasted 30–60 min, with most lasting approximately 45 min. All interviews were audio-recorded and transcribed.

### Analysis

Participant characteristics were quantitatively summarized. Transcripts were qualitatively analyzed using inductive content analysis, [36] in which codes were derived from the data and added to the codebook as transcripts were analyzed. All transcripts were independently coded by two analysts with experience in qualitative analysis and substance use-related research (MCF, EMS). The analysts met regularly to review each coded transcript, resolve discrepancies, and add to/refine the codebook as needed. Transcripts were coded using Atlas.ti 22 software [37]. Data collection continued until analysts agreed that saturation of themes had been reached among the entire sample; [38, 39] at this point recruitment of primary care providers ended, but we continued our attempts to recruit all eligible non-primary care providers to increase representation of other settings. Codes and example quotations were iteratively reviewed by the full investigative team to ensure that themes were supported by the data and finalize themes by consensus. Factors impacting MOUD receipt for patients with co-occurring substance use were organized under TICD checklist domains [34].

## Results

### Sample description

Twenty-seven providers participated in interviews, a 30% response rate among 90 providers who were contacted (this rate excludes 9 providers who responded but did not meet eligibility criteria). Participant characteristics are presented in Table 1. Just under three-quarters of participants provided buprenorphine care at a VA medical center (larger facilities that provide a wider range of general and specialty services), and just under half provided buprenorphine care at one or more community-based outpatient clinics (smaller facilities that provide primary care and other common outpatient services; types of services provided vary across clinics) [40]. Most provided buprenorphine care in urban locations, and over one-third provided buprenorphine care in rural locations [41]. The most common clinical setting was primary care, followed by mental health and pain (pharmacists supported care across multiple clinical settings). The most common clinical training was physician, followed by nurse practitioner/physician assistant and pharmacist. Most participants had completed buprenorphine waiver training outside of the VA, and most had received some other type of MOUD education (e.g., in residency, VA meetings or trainings). Participants had been in their current position for an average of 3.5 years and had worked at the VA for an average of 6.9 years. They had provided buprenorphine care for an average of 4.9 years (ranging from 8 months to 16 years) and were currently providing buprenorphine care for an average of 20.4 patients (ranging from zero to 80).

**Table 1:** Interview participant characteristics (N = 27)

	N	%
VA workplace type(s)^a^		
VA medical center (VAMC)	14	52
Community-based outpatient clinic (CBOC)	7	26
Both VAMC and CBOC	6	22
VA workplace location(s)^a^		
Urban	17	63
Rural	6	22
Both urban and rural	4	15
Clinic type		
Primary care	12	44
Mental health	7	26
Pain	4	15
Pharmacist (multiple clinic types)^b^	4	15
Clinical training		
Physician	16	59
Nurse practitioner/physician assistant	7	26
Pharmacist	4	15
Buprenorphine waiver training^c^		
Completed outside of VA	21	78
Completed through VA	5	19
Did not complete	1	4
Addiction certification/fellowship	3	11
Received other MOUD education^d^		
Yes	21	78
No	6	22

	Mean (SD)	Range
Years in current position	3.5 (2.6)	8 months–10 years
Years at VA	6.9 (5.7)	8 months–24 years
Years providing buprenorphine^e^	4.9 (3.3)	8 months–16 years
Estimated # of patients currently prescribing buprenorphine for^d^	20.4 (22.6)	0–80 patients

*MOUD* medications for opioid use disorder; *VA* Veterans Health Administration

^a^Some participants provided buprenorphine care at multiple VA facilities

^b^Pharmacists supported buprenorphine care in multiple clinic settings

^c^At the time of this study, pharmacists could not obtain a waiver or prescribe buprenorphine, but some had completed the waiver training

^d^Types of other MOUD training included training during residency; grand rounds and case review meetings; continuing education courses; VA conferences, trainings, and implementation interventions

^e^Missing for n = 1 participant

### Approaches to addressing OUD in patients with co-occurring substance use

#### Perceptions of co-occurring substance use among patients with OUD

Most participants reported that co-occurring substance use was common among their patients with OUD. Alcohol and cannabis were frequently described as the most common substances, and some participants reported that methamphetamine use was also common or increasing. Benzodiazepines, cocaine, and other drugs were less frequently mentioned. Some participants contrasted patients who use illicit opioids to those who use prescription opioids, perceiving that the former were more likely to use other illicit substances.

#### Assessment of co-occurring substance use among patients with OUD

Participants typically assessed other substance use through patient self-report (e.g., “I always ask the patient at my initial assessment, of every history of substance use that they’ve had, and if they’re using any.” [P13, pharmacist, both urban and rural]) and/or biological tests (e.g., urine drug screens). Less common approaches included administering standardized assessments of substance use (e.g., the Alcohol Use Disorder Identification Test-Consumption screen) [42] or reviewing health record information (e.g., chart notes, documented SUD diagnoses, prescribed medications). Participants expressed mixed opinions on the value of urine drug screens—some felt they were useful for obtaining more objective information and discouraging other substance use, while others felt repeated testing could be detrimental to their relationship with the patient.

“*I think urine drug screens are a deterrent for them to go out and seek other drugs*.” [P2, pain, nurse practitioner, urban]

“*I think building rapport with the patient in serial interviews about their relationship with other substances, I find that to be more effective. Particularly because for some individuals, providing urine samples was associated with a punitive system, and it can be detrimental to serially test someone, detrimental to your therapeutic alliance*.” [P1, primary care, physician, urban]

#### Providing OUD treatment to patients with other substance use

Many participants reported that they prescribed buprenorphine or managed buprenorphine care for patients with co-occurring substance use. Most recommended additional services to address other substance use (e.g., treatment for other SUDs, mutual support groups). Some also provided medications for co-occurring alcohol use disorder; these participants noted that buprenorphine and naltrexone cannot be used at the same time, and some reported that they typically prioritized buprenorphine and prescribed alcohol use disorder medications other than naltrexone. Others provided injectable naltrexone instead of buprenorphine to simultaneously treat co-occurring OUD and alcohol use disorder, though some believed naltrexone was a less effective OUD treatment and required high patient motivation.

“*If somebody is really motivated to treat both alcohol and opioid use disorder, [injectable] naltrexone is a great option*.” [P8, mental health, physician, urban]

Some participants described additional measures they took when providing buprenorphine to patients with other substance use, including educating patients about potential risks, motivational interviewing to encourage reduction or cessation, and increasing monitoring through higher frequency of visits, shorter refill periods, and/or increased screening for substance use. Some reported that they sometimes prescribed a lower dose of buprenorphine to patients with co-occurring substance use due to concern about respiratory depression.

“*…we might…lower the [buprenorphine] dose if they’re on max dose and we’re worried about respiratory depression*.” [P12, pharmacist, both urban and rural]

Another participant said they increased the dose for patients with co-occurring methamphetamine use due to concerns about fentanyl contamination.

“*With my patients that are actively using meth, given the amount of fentanyl that is in meth, [I] do try to have them on higher doses. I think there is some data that there is a decrease in meth use at higher doses of buprenorphine*.” [P23, primary care, physician, urban]

Although many participants in this study provided buprenorphine to patients with other substance use, several perceived that most VA buprenorphine providers outside of SUD specialty settings do not:

“*Most providers that I work with outside of [SUD] specialty care do not touch patients that use other substances. And they do not start buprenorphine in those patients*.” [P13, pharmacist, both urban and rural]

#### Referring to SUD specialty care for OUD treatment

Several participants reported referring patients with other substance use to specialty SUD settings for OUD treatment, either for any level of use or higher severity use. Some were willing to provide a short-term prescription until the patient was able to start SUD specialty care or would consider providing buprenorphine if the patient was unwilling to go to the SUD clinic, but others indicated they would not initiate or continue prescribing buprenorphine for these patients.

“*If it’s someone who is not going to abstain from alcohol use, or not wanting treatment for that and is heavily using, that’s someone we would not consider starting in the primary care setting, that’s someone who needs to be seen in the [SUD specialty clinic]*.” [P15, primary care, nurse practitioner, urban]

### Factors impacting MOUD receipt for patients with co-occurring substance use

Factors impacting MOUD receipt are organized under the TICD Checklist domains and summarized in Table 2.

**Table 2:** Factors impacting MOUD receipt for patients with co-occurring substance use, organized by TICD Checklist domains

**Individual health professional factors**
*Providers’ awareness of recommendations*
•In general, participants reported that there are not clear recommendations around buprenorphine care for patients with co-occurring substance use in guidelines or training •Other information sources may shape providers’ approaches (e.g., colleagues, doing their own research) •Participants generally agreed that patients using other substances should receive MOUD, but varied in how they viewed their primary role (i.e., providing the care vs. facilitating linkage to higher-level care)
*Other individual health professional factors*
•Some providers may lack relevant knowledge/skills/experience •Providers have a range of perceptions/attitudes that may impact their approach (safety/other concerns; beliefs about appropriateness of non-SUD care setting; harm reduction philosophy; patient-centered approach)
**Providers’ perceptions of patient factors**
•Life instability related to co-occurring substance use may create barriers to receiving MOUD care •Fear of disclosing co-occurring substance use may be a barrier to receiving MOUD care •Patients may or may not prefer to receive MOUD in an SUD specialty setting, which may be impacted by addiction-related stigma
**Professional interactions**
•Collaboration with SUD experts may facilitate buprenorphine provision for patients with co-occurring substance use outside of SUD specialty settings, or facilitate linkage to SUD specialty care •Siloed care/expertise may make it more difficult to adequately support these patients •Existing VA efforts to integrate primary care and mental health may not adequately address SUD care
**Incentives and resources**
*Within participants’ clinics*
•Lack of adequate time with patients to address complex issues may be a barrier •Lack of nursing and other staff may be a barrier
*Outside participants’ clinics*
•Low accessibility of SUD specialty clinics may be a barrier to linking patients to higher-level MOUD care and/or additional care for other SUDs •Availability of other higher-level SUD care (e.g., detox, residential treatment) may be too low •Mental health and social services provided though the VA may help patients with co-occurring substance use engage in MOUD care, but there may be barriers to accessing these services
**Capacity for organizational change**
•Clinic policies/treatment agreements banning other substance use may have become more flexible in recent years to encourage increased provision of buprenorphine •Some SUD specialty clinics may still have strict rules around other substance use or generally require more structured care, which may present barriers for some patients with co-occurring substance use •Leadership in primary care, mental health and pain clinics may vary in their support of buprenorphine provision for this population
**Social, political and legal factors**
•Telehealth does not seem to greatly impact providers’ approach to treating OUD among patients with co-occurring use, but may make it more difficult to assess other substance use •Telehealth may have increased access to SUD specialty services for some patients in rural areas, however the COVID-19 pandemic may have also decreased provision of these services •Cannabis legalization/normalization may have made some providers more willing to provide buprenorphine care to patients who use cannabis •Concerns about overdose risk related to a rise in fentanyl use may increase providers’ sense of urgency of providing buprenorphine regardless of other substance use

*MOUD* medications for opioid use disorder, *OUD* opioid use disorder, *SUD* substance use disorder, *TICD* Tailored Implementation for Chronic Diseases, *VA* Veterans Health Administration

#### Providers’ awareness of recommendations

In general, participants reported that there are not clear recommendations around buprenorphine care for patients with co-occurring substance use in guidelines or training. About half of the participants were not familiar with formal clinical guidelines related to this issue, and some noted that providers outside of SUD specialty settings may be less likely to be aware of these guidelines compared to SUD specialists.

“*I should know…if there is [a guideline] I would imagine it’s provided to the [SUD specialty clinic] staff, but not necessarily to primary care providers*.” [P15, primary care, nurse practitioner, urban]

Similarly, most participants said this topic was not addressed in training they had received on buprenorphine care, or that they did not recall if it was addressed.

“*I went back and looked at the buprenorphine waiver training…no detailed information that I could really easily come up with for clarification or guidance on what’s the best way to manage patients who are not interested in giving up methamphetamines*.” [P17, mental health, nurse practitioner, rural]

Those who did recall specific recommendations from guidelines or training described varied content, including assessing for other substance use, providing buprenorphine regardless of other substance use, being aware of potential risks and using caution when prescribing, increasing monitoring, and referring patients with other substance use to SUD specialty care rather than prescribing.

“*They say…you give the buprenorphine if [patients] need the buprenorphine, even if they are using other substances. I think I’m pretty much in line with what they’ve been telling me on the teleconferences and the courses I’ve been taking*.” [P7, pain, physician, urban]

“…*there wasn’t a lot of discussion [in trainings] about concomitant treatment of opioid dependence and alcohol use disorder. I think the recommendation was to move them onto a more specialized care setting*.” [P4, primary care, physician, urban]

Despite reporting low familiarity with formal guidelines and inconsistent training, participants generally felt that patients should receive MOUD in the presence of other substance use. Some viewed their primary role as providing this care, while others viewed their primary role as facilitating linkage to OUD care in SUD specialty settings. Participants described other sources of information that shaped their understanding of OUD care for patients with co-occurring substance use, including modeling their practice after other providers in their facility, consulting with colleagues (including SUD specialists), and doing their own research.

“*I think most of my practice is more informed by just discussion with colleagues who are specialty trained about how they handle situations*.” [P20, primary care, physician, urban]

#### Other individual health professional factors

Participants also described a range of perceptions and attitudes (both their own and their perceptions of other providers) that may influence approaches to addressing OUD in patients with co-occurring substance use. Many participants had safety concerns about combining buprenorphine with other substances, which sometimes led to extra precautions (e.g., increased monitoring) or referral to an SUD specialty setting for OUD care. Most were specifically concerned about alcohol and benzodiazepines because they may increase risk of respiratory depression.

“*The biggest concern is if they’re using something else that increases risk of respiratory depression…like benzodiazepines or alcohol, I’d be much more concerned than with cannabis use.”* [P10, mental health, physician, urban]

However, some providers expressed more concern about “illicit” substances.

“*If a veteran ever popped for cocaine, amphetamines, things like this, then that would be immediately a [SUD specialty clinic] situation…Because…they’re illicit substances. There’s already a component of, I’m doing something illegal, unsafe*.” [P16, pain, physician assistant, urban]

Participants also described other concerns, including other substance use interfering with adherence to buprenorphine and diversion of buprenorphine to obtain other substances.

“…*there might be some concern for diversion for patients who are using other substances…like diverting half of it in order to obtain money for whatever their other substance of choice is*.” [P12, pharmacist, both urban and rural]

Many participants across all clinic settings endorsed a “harm reduction” philosophy of buprenorphine provision emphasizing that it is more dangerous to let OUD go untreated than to prescribe buprenorphine to patients with co-occurring substance use. This belief was usually tied to a willingness to prescribe to these patients outside of SUD specialty settings.

“*I think from a harm reduction standpoint, regardless of other substance use, patients should be offered treatment to lower the risk of adverse outcomes from opioid use, including overdose. Because we know that buprenorphine reduces that risk.”* [P6, primary care, physician, urban]

One participant emphasized the role of this philosophy in driving variability in individual providers’ approaches.

*“…there is a sort of a divide between…prescribers who really embrace a harm reduction philosophy and those who don’t…even within our group there are providers who feel more or less willing to continue prescribing if someone is using another substance*.” [P20, primary care, physician, urban]

For some participants, a desire to respect patients’ preferences was another driver of providing buprenorphine for these patients outside of an SUD specialty setting.

“…*there’s a good percentage of patients who would prefer to receive this care in primary care, and we wouldn’t be meeting the needs of those individuals if we mandate that they receive specialist treatment because they’re using other substances*.” [P1, primary care, physician, urban]

Alternately, participants perceived that many providers outside of SUD specialty settings do not prescribe buprenorphine to patients using other substances due to a belief that SUD specialty care is the only appropriate treatment setting for this patient population.

“*I think that…general practitioners are uncomfortable prescribing buprenorphine to people who don’t look ‘perfect,’ right? So, if you’re sort of a ‘white collar’ individual who has an opioid use disorder because of overuse of pain medication, then they’re willing to prescribe buprenorphine. But if you’re somebody with other co-occurring substances they think that you need to be in an addiction treatment center, or are unwilling to prescribe you buprenorphine, or both*.” [P11, mental health, physician, urban]

Participants also described how lack of knowledge, skills and experience related to treating patients with polysubstance use among some providers could prevent or delay buprenorphine care.

“*I think it has to do with provider awareness and knowledge about how to treat patients that are more complex…Instead of doing…what they think might be the wrong thing, they may just say, ‘sorry you’ve got to go through withdrawals, I can’t prescribe because I’m not comfortable, you have to wait until this consult service calls.’ So, it’s like a delay in care*.” [P14, pharmacist, both urban and rural]

#### Providers’ perceptions of patient factors

Participants perceived that instability in patients’ lives related to other substance use created barriers to engaging in MOUD care in both their clinic and SUD specialty settings. They described several sources of instability including intoxication and withdrawal, mental health conditions, strained relationships, unemployment, housing instability, and legal system involvement.

“*The danger is not necessarily the substance itself, but the chaos it creates in their life, physical and mental health, and socially as well…Their ability to come in for appointments is all over the place*.” [P5, primary care, physician, urban]

Participants also perceived that fear of disclosing other substance use may present a barrier to OUD care for patients with co-occurring substance use.

“*[Patients] may be less likely to try and get [OUD] care if they know that they’re using illicit substances, they feel like they’ll be in trouble. So, they just don’t try to get care.*” [P14, pharmacist, both urban and rural]

When discussing referring patients with co-occurring substance use to SUD specialty care, participants discussed their perceptions of how stigma might impact some patients’ preferences around the setting in which they receive MOUD care. Some perceived that many patients see SUD specialty settings as stigmatizing and therefore prefer to receive treatment in other healthcare settings.

“*A lot of patients will do everything they can to avoid the [SUD specialty clinic]…Due to the stigmatization, they’d rather be treated outside of there if they can*.” [P7, pain, physician, urban]

However, one participant perceived that some patients experience less stigma in SUD specialty care compared to primary care.

“*I would imagine that they would prefer specialty care just because the people that are in specialty care have worked with patients similar to them. So that understanding is there. I hear a lot from patients that their primary care provider doesn’t understand, they’re treating them like an addict*.” [P26, pharmacist, rural]

#### Professional interactions

Participants described a broad spectrum of cross-disciplinary collaboration and discussed how varying degrees of collaboration impacted their approach to MOUD care. Some participants had clinicians with SUD expertise integrated into their clinic or had regular close collaboration with them—for example, collaboration between clinicians prescribing buprenorphine outside of SUD specialty settings and SUD clinical pharmacy specialists who manage the care, or regular meetings including SUD specialty care, primary care, mental health, and/or pain providers to discuss complex cases. This type of collaboration was reported by participants from larger and smaller as well as urban and rural facilities. Some participants discussed how collaboration and access to SUD expertise facilitated buprenorphine provision for patients with co-occurring substance use outside of SUD specialty settings.

“*It depends on how actively [providers are] engaged in kind of a collegial, collaborative process around care…Some people may have kind of a sharp line over which they don’t prescribe [buprenorphine] in circumstances. And then others who work more with the [SUD specialty clinic] in a collaborative fashion I think are willing to have more flexibility with their prescribing. I think having support for the individual provider from more experienced clinicians is pretty critical to create some more flexibility in that care*.” [P4, primary care, physician, urban]

Other participants described how cross-disciplinary collaboration led to improved handoffs to SUD specialty care rather than increased buprenorphine prescribing in their clinical setting.

“*I feel like one of the things that makes it easier is that we have kind of a whole system where we can do a warm handoff to addiction services…we can kind of work in concert. I know I did not have that when I worked in private, I would just have to recommend the referral, and then…I don’t even know what would happen, if they went, or were getting the care they needed*.” [P16, pain, physician assistant, urban]

Alternately, some participants at both larger and smaller as well as urban and rural facilities reported that care and expertise was siloed between disciplines, which made it more difficult to adequately support patients with co-occurring substance use.

“*Having access and really being involved in the substance use clinic, and having a counselor, is what my goal would be [for patients with co-occurring substance use]. But I have to say that I don’t have much of an awareness or a relationship with how that system runs. Mental health seems to be quite separate from primary care here*.” [P19, primary care, physician, rural]

Several primary care providers felt that the VA’s Primary Care-Mental Health Integration model (PCMHI), a national effort to formally integrate mental health into primary care, did not adequately support substance use-related care.

“…*not every PCMHI person is also comfortable with opioid use disorder, or substance use disorder. So, if we’re talking about obstacles, that would be another piece to improve…their knowledge of substance use disorder, since they’re kind of connected in the realm of primary care*.” [P3, primary care, nurse practitioner, both urban and rural]

Some participants providing care at rural facilities reported that they were the only buprenorphine provider in their clinic. These participants were prescribing buprenorphine to patients with co-occurring substance use, suggesting that this isolation was not necessarily a barrier to doing this. However, some noted they would like to have more information about what other providers are doing.

“*I think that maybe I’m not too far off what everybody else does. I would be curious to see how I pair up.”* [P25, primary care, nurse practitioner, rural]

#### Incentives and resources

Participants reported a lack of resources within their clinic needed to provide MOUD to patients with co-occurring substance use, including nursing staff, support personnel, and lack of adequate time to address more complex issues with patients. Some described how turnover contributed to lack of time and staff.

“*They want me to see patients every half hour, and some of these patients you need an hour with, because of the issues they have. You can’t deal with alcohol and opioid use disorder…in half an hour*.” [P7, pain, physician, urban]

Participants also discussed resources outside of their clinic. The accessibility of SUD specialty clinic services was described as an important barrier or facilitator to caring for patients with co-occurring substance use, with respect to referring patients there for MOUD and/or for additional services to address other SUDs. Many mentioned specific barriers including far distance, limited hours of availability, and wait times. Alternately, some participants in urban facilities described these services as more accessible when they were located at the same facility as their clinic and had same-day access.

“*…the VA serves a population in our [VA regional network] that resides in a very large geographic area. Yet we provide all of our specialty services in two cities that are very difficult for people to access…there are a lot of individuals who would like to receive [SUD specialty clinic] services and cannot because of their geographic location*.” [P1, primary care, physician, urban]

Some participants also described the low availability of other higher-level SUD services needed to support some patients with co-occurring substance use, including detoxification services and residential treatment.

“*We need more support at the higher levels of care for people, like some of these really complex patients…I think if it was easier for them to access residential treatment that could go a long way. I think the front door is more open than it used to be, people can walk in and get linked to care, but if their trajectory is not successful…it’s almost like we don’t have enough care at the higher intensity level*.” [P20, primary care, physician, urban]

Finally, some participants described how the availability of other VA services, including mental health and social services, helped patients with co-occurring substance use engage in MOUD care.

“*The VA makes it so much easier to care for patients as compared to the community*…*Our ability to provide housing, and potentially employment, probably has the biggest impacts on our ability to help someone manage their conditions…That’s the thing that sets the VA apart*.” [P5, primary care, physician, urban]

However, some mentioned barriers to accessing these services including distance and wait times for mental health services (particularly in rural facilities) and complex processes for signing up for social services.

#### Capacity for organizational change

Participants discussed how policies in primary care, mental health and pain clinics impacted MOUD care for patients with co-occurring substance use. Some participants in rural facilities reported that their clinics employed OUD treatment agreements that require or strongly recommend abstinence from other substances, but that the language and/or enforcement had become more “lenient” in recent years.

“*The old protocol said, ‘I will not abuse any substances.’ And it has a place for the patient to initial. I think the new one says that we recommend not abusing any of the other substances, the verbiage has just become more lenient*.” [P26, pharmacist, rural]

Some linked changes in their clinic’s policy to broader policies encouraging expanded provision of buprenorphine.

“*Originally [no substance use] was part of the contract for treatment. I think it has to do with the changes that have been happening on a bigger scale. It seems like everything that’s coming through, whether it’s local policy, or national policy, there’s just a lot of encouragement for [buprenorphine] to be more widely available, to be available in a variety of different settings, and to get it to people because it seems to really work*.” [P17, mental health, nurse practitioner, rural]

Participants also discussed the impact of clinic leadership. One participant felt that leadership in their clinic did not support buprenorphine provision for people with co-occurring substance use.

“*I feel pressured sometimes, ‘Dr. [name], what are you doing prescribing buprenorphine to this person when they have this urine drug screen for amphetamines?’* ” [P24, pain, physician, urban]

Alternately, another participant described how their current VA clinic was more supportive of treating patients with co-occurring substance use compared to the lack of support from leadership in a non-VA setting they had previously worked in.

“*The last place I worked at before coming here…one of the reasons I left was because the administration was saying if they use meth you have to cut them off, if they use benzos, you have to cut them off…frankly, the reason I’m at the VA is because I refused to stop treating patients with polysubstance use*.” [P23, primary care, physician, urban]

Several participants reported that some SUD specialty clinics have restrictive policies around other substance use (e.g., requiring negative urine drug screen results to receive MOUD), or generally require more structured care (e.g., more frequent visits). Some discussed how these restrictions might result in patients with more complex needs who have “stepped up” to receive care in an SUD specialty setting being lost to follow-up or “stepping off” (i.e., ending up in a lower-level setting for MOUD care).

“*In the VA we have the ‘stepped care’ model for opioid use disorder…we’re like when people just ‘step off.’ That’s some of our patients…I wonder if that’s because in other settings they tend to be more regulated…and more restricted. So, I wonder if [more regulated SUD specialty care] just doesn’t work for some people, and so that’s why we end up seeing them in our sort of catch-all clinic*.” [P20, primary care, physician, urban]

#### Social, political and legal factors

Finally, participants described factors external to the VA healthcare system impacting MOUD care for patients with co-occurring substance use. Many participants were providing buprenorphine via telehealth due to the COVID-19 pandemic and reported that telehealth generally did not change their approach to treating patients with co-occurring substance use. However, some reported that it was more challenging to assess for other substance use via telehealth.

*“[Telehealth] doesn’t really impact too much the decision to prescribe buprenorphine [to patients with other substance use], we just have to…ask more questions, ask the same questions in different ways, to try to make sure that you’re getting…as full of an assessment as you can get via phone or via video*.” [P14, pharmacist, both urban and rural]

Some participants said that telehealth had increased access to specialty SUD services for patients in rural areas but noted limitations, including some patients’ lack of internet/phone access or preference for in-person care. However, some reported that the pandemic had negatively impacted availability of SUD services (e.g., reduced provision of SUD specialty clinic services and residential treatment).

Participants also described how changes in the substance use landscape had impacted MOUD care for patients with co-occurring substance use. Some discussed how the legalization and normalization of cannabis use had made them more willing to prescribe buprenorphine for patients who use cannabis, but this change was challenging for some.

“*I was trained in a world where cannabis was an illegal substance…if I were to use the standards that I was trained under, then I would have to kick out…the majority of my patients. So it is, that is a challenge*. *I feel like I’ve had to be flexible to explain the risks to their long-term goals in their life. And balance that with the stability that they’re getting with [buprenorphine]*.” [P18, mental health, physician, urban]

Participants also believed that a sharp increase in fentanyl use, and other opioids being contaminated with fentanyl, had increased risk of overdose for their patients with OUD. They discussed how this made a harm reduction approach to buprenorphine provision in the presence of co-occurring substance use more urgent.

*“[Prescribing buprenorphine is] definitely safer than having them continue to use opioids that they’re getting off of the street, especially with the increase in the amount of opioids on the street that…are contaminated with fentanyl*.” [P6, primary care, physician, urban]

### Support needed to increase MOUD receipt among patients with co-occurring substance use

Participants were asked what support they felt was needed to increase MOUD receipt for patients with co-occurring substance use. Support recommended by participants is summarized in Table 3.

**Table 3:** Support recommended by participants to increase MOUD receipt among patients with co-occurring substance use

•Create clear institutional policies/guidelines related to providing buprenorphine to patients with co-occurring substance use •Provide more specific education/training to providers outside of SUD specialty settings related to treating OUD among patients with co-occurring substance use •Increase collaboration between buprenorphine providers outside of SUD specialty settings and SUD specialists •Give buprenorphine providers outside of SUD specialty settings more time to spend with each patient •Increase nursing and other staff •Increase same-day availability •Increase availability of SUD specialty services •Improve linkage to SUD specialty services (integrate SUD care into other settings, co-locate SUD clinics with other clinics, improve referral and warm hand-off processes)	

*MOUD* medications for opioid use disorder, *OUD* opioid use disorder, *SUD* substance use disorder

Participants recommended providing education/training to providers outside of SUD specialty settings related to treating OUD among patients with co-occurring substance use. Some suggested specific content including monitoring, buprenorphine dosing, providing injectable vs. oral/sublingual buprenorphine, referral, and motivational interviewing. They also suggested providing data on the risk of death associated with providing vs. not providing buprenorphine when patients are using other substances, and education on harm reduction principles.

“*Maybe some data for data-driven folks, this is the risk of death off buprenorphine for these folks, this is the risk of death with buprenorphine… Because I think people are just so scared to cause harm. And some of the decisions are just about weighing pros and cons, and I think people just do it incorrectly sometimes*.” [P23, primary care, physician, urban]

Participants also recommended increasing collaboration between clinicians providing buprenorphine care outside of SUD specialty settings and SUD specialists. Suggestions included having experts available for consultation and regular meetings to discuss cases.

“*We have wonderful conferences that anybody can pop into to discuss a case…a group of colleagues meeting regularly where you can just be like, ‘Hey, what do you do with this situation that I’ve never encountered before?’ Everyone should have that*.” [P8, mental health, physician, urban]

Participants recommended giving clinicians who are providing buprenorphine care outside of SUD specialty settings more time to spend with each patient, increasing nursing and other staff in their clinic, and increasing same-day access in their clinic. They also recommended increasing the availability of SUD specialty services and improving linkage to these services through integrating more SUD care into other care settings, locating SUD clinics at the same physical location as other clinics, and improving referral and warm hand-off processes.

“*Maybe co-locating [SUD specialty] treatment programs within primary care. So that stigma goes away, it’s more normalized again*.” [P3, primary care, nurse practitioner, both urban and rural]

Finally, a few participants recommended creating clear institutional policies or guidelines related to providing buprenorphine to patients with co-occurring substance use. They discussed how this would help providers feel more supported in taking a harm reduction-informed and patient-centered approach to care.

“*Maybe it would be policy. It would be encouraging to know that, as a provider, if you’re taking this harm reduction approach, that you’re supported. Because it’s scary and it’s risky. But we’re essentially meeting patients where they are, as opposed to demanding that they meet our requirements in order to have access to a life-saving treatment…just knowing that’s a decision, that’s an approach that would be supported*.” [P17, mental health, nurse practitioner, rural]

## Discussion

This qualitative study examined the experiences and perspectives of VA clinicians providing buprenorphine care in primary care, mental health, and pain clinics related to addressing OUD among patients with co-occurring substance use. Although they reported that this topic was not clearly addressed in clinical guidelines or training, participants generally felt that patients should receive MOUD in the presence of other substance use. Some viewed their primary role as providing this care, while others viewed their primary role as facilitating linkage to OUD care in SUD specialty settings. Participants described multiple barriers and facilitators to providing buprenorphine to patients with co-occurring substance use, as well as barriers and facilitators to linking them to SUD specialty care.

Consistent with the concept of stepped care, patients with OUD who have co-occurring substance use may benefit from receiving MOUD in higher-intensity (e.g., SUD specialty care) rather than lower-intensity (e.g., primary care) settings, particularly those with co-occurring SUDs or who are actively using alcohol or sedatives [20, 43]. However, participants in this study pointed out multiple reasons why many of these patients may not initiate or be retained in MOUD care in SUD specialty settings. As non-SUD specialty settings may be the only viable option for MOUD care for many patients, [19] it is important that clinicians providing this care outside of SUD specialty settings are supported in caring for patients with co-occurring substance use. Although many participants in this study prescribed buprenorphine for this patient population, they perceived that most providers outside of SUD specialty settings do not and described multiple barriers.

In this study, we broadly asked participants about treating patients with co-occurring substance use. Most participants did not clearly distinguish between co-occurring substance use and co-occurring SUDs when describing their approach to MOUD care (whether they tended to provide MOUD or refer the patient to specialty SUD care for MOUD in the presence of other substance use). When asked how they assessed other substance use, participants did not describe using structured SUD assessments to identify co-occurring SUDs, and rarely described reviewing the medical record for SUD diagnoses (though these methods of assessment were not asked about directly). Thus, providers may benefit from training on how to assess for the presence and severity of co-occurring SUDs and implications for MOUD care. Pragmatic SUD assessment tools, such as symptom checklists, may help MOUD providers regularly assess for co-occurring SUDs as is recommended in clinical guidelines [44–46]. Additionally, participants in this study reported varied approaches to assessing co-occurring substance use among patients with OUD. Using validated screening tools to assess for other substance use is recommended for office-based MOUD care, [47] and MOUD providers may benefit from access to and training in using these tools.

Findings suggest that clinical guidelines related to providing buprenorphine to patients with co-occurring substance use should be made more visible to clinicians providing this care outside of SUD specialty settings, and that these clinicians may benefit from more detailed and directive guidance. Many providers may not be familiar with national guidelines or may find them vague. Specific, consistent and clear guidelines/policies communicated at the clinic or facility level may help providers feel more comfortable and supported in caring for patients with co-occurring substance use.

Participants also noted the need for specific training on how to most effectively treat OUD in patients with co-occurring substance use (e.g., monitoring, dosing), and suggested that providers outside of SUD specialty settings may benefit from education on the relative harms of providing vs. not providing buprenorphine and harm reduction principles. This information could be more systematically included in buprenorphine trainings and continuing education materials. As some providers may gain more information from interactions with colleagues and experience treating patients compared to reading clinical guidelines or attending trainings, these concepts could also be integrated into multiple care improvement activities such as cross-disciplinary meetings or clinical decision-support tools. Efforts are needed to improve the integration of SUD care and expertise in non-SUD specialty settings, such as increasing capacity to provide SUD care in PCMHI clinics, [48] testing collaborative care models for patients with polysubstance use, and creating other opportunities for buprenorphine providers in these settings to collaborate with SUD specialists. Additionally, lack of adequate provider time and staffing, consistently reported as barriers to expanding MOUD care, [49, 50] remain important problems to be addressed.

Simultaneously, efforts are needed to improve access to SUD specialty clinics and other higher-level SUD care (such as supervised detoxification and residential treatment) for patients who would benefit from and are willing to receive this care. These efforts may include devoting funding and resources to provide these services in more locations and during expanded hours, as well as clarifying referral pathways and creating warm handoff processes. Increased telehealth provision of SUD specialty treatment may increase access for patients living far from VA facilities, and work is needed to continue assessing and improving the quality of this care [51, 52]. Co-locating SUD specialty services with other clinics and use of warm handoffs may facilitate linkage and reduce stigma for patients. Finally, SUD specialty clinics should adopt flexible policies around providing MOUD to patients with ongoing substance use (e.g., not requiring abstinence from other substances to receive MOUD) and may consider other ways to increase flexibility in care for patients who struggle to meet clinic requirements.

These findings should be considered in the context of research assessing perspectives on MOUD access among people who use multiple substances. People with polysubstance use have reported that it can worsen health and social situations, [53] which may create barriers to treatment, and have reported being discharged from MOUD treatment due to other substance use, [54] barriers that align with providers’ perspectives in this study. People who use both methamphetamine and opioids have reported feeling a “balancing” effect of the two drugs that improves their functioning, [54, 55] which for some might decrease interest in receiving MOUD treatment, and this is a perspective that providers may be less aware of. Further research assessing the perspectives VA patients who have OUD and co-occurring substance use is needed.

This study has strengths and limitations. While a qualitative approach allowed us to obtain rich, detailed information and discover unanticipated factors affecting this care, findings cannot be considered representative of all buprenorphine providers in the regional network given the response rate of 30%. Specifically, providers who agreed to participate may have been more willing to provide buprenorphine care to patients with co-occurring substance use than those who did not participate, a possibility supported by the finding that many participants perceived that most other providers outside of SUD specialty settings were not willing to prescribe for these patients. This study was also limited by low representation of certain groups with a smaller number of eligible providers, which prevented us from systematically assessing differences in themes across clinical settings and provider characteristics. Primary care, mental health, pain, and pharmacy settings differ in their practice structure, clinical scope, and training, and further qualitative research with larger and more evenly balanced samples is needed to rigorously assess potential differences in barriers and facilitators across settings and provider characteristics. Three participants in this sample had an addiction certification or addiction fellowship training, and future research should assess whether there are differences for providers with these credentials compared to those without. Findings from this study could also inform future quantitative surveys that can compare the prevalence of different barriers and facilitators overall and across different clinic settings and provider characteristics. Asking broadly about co-occurring substance use limited our ability to understand whether providers’ approaches and perceptions of barriers and facilitators differ for patients with co-occurring SUDs vs. lower severity use, or for patients with co-occurring use of different substances, and future research is needed that more precisely examines these questions. Finally, findings may have limited generalizability in non-VA healthcare settings and in other regions of the country, which may differ with respect to relevant policies (e.g., cannabis legalization), the drug supply (e.g., prevalence of fentanyl), and the epidemiology of substance use and overdose.

## Conclusions

In this qualitative study of 27 VA clinicians providing buprenorphine care in primary care, mental health and pain clinics, participants reported varied approaches to assessing other substance use and varied approaches to treating OUD for these patients. Participants reported multi-level barriers and facilitators to providing buprenorphine care to patients with co-occurring substance use, as well as barriers and facilitators to linking these patients to care in SUD specialty settings. Specifically, they reported a lack of clear recommendations in guidelines and training, and discussed their perceptions of how provider factors (e.g., knowledge and attitudes), patient factors (e.g., life instability related to other substance use and preferences around treatment setting), organizational factors (e.g., cross-disciplinary collaboration, resources, and clinic policies/leadership), and external factors (e.g., the COVID-19 pandemic and changes in the substance use landscape) impacted MOUD receipt for these patients. The majority of people with OUD use other substances, and the VA and other healthcare systems need to address barriers to MOUD for these patients. Efforts are needed to support clinicians outside of SUD specialty settings in providing buprenorphine care to patients with co-occurring substance use, as well as to improve linkage to SUD specialty clinics and other higher-level SUD care. These efforts may increase MOUD receipt and improve OUD care quality for patients with co-occurring substance use.

## Data Availability

Data are not publicly available due to institutional rules regarding data sharing.

## Abbreviations

OUD: Opioid use disorder
MOUD: Medications for opioid use disorder
SUD: Substance use disorder
TICD: Tailored Implementation for Chronic Diseases
VA: Veterans Health Administration
